# m^6^A regulator-mediated methylation modification patterns and tumor immune microenvironment in sarcoma

**DOI:** 10.18632/aging.203807

**Published:** 2022-01-03

**Authors:** Zhehong Li, Junqiang Wei, Honghong Zheng, Xintian Gan, Mingze Song, Yafang Zhang, Lingwei Kong, Chao Zhang, Jilong Yang, Yu Jin

**Affiliations:** 1 Traumatology and Orthopedics, Affiliated Hospital of Chengde Medical College, Chengde, Hebei 067000, China; 2 Department of Bone and Soft Tissue Tumor, Tianjin Medical University Cancer Institute and Hospital, Tianjin 300060, China; 3 National Clinical Research Center for Cancer, Key Laboratory of Cancer Prevention and Therapy, Tianjin’s Clinical Research Center for Cancer, Tianjin 300060, China; 4 Department of Orthopedics, Affiliated Hospital of Chengde Medical College, Chengde, Hebei 067000, China; 5 General Surgery, Affiliated Hospital of Chengde Medical College, Chengde, Hebei 067000, China

**Keywords:** m^6^A modifications patterns, m^6^A regulators, tumor immune microenvironment (TIME), sarcoma

## Abstract

Background: Studies have shown that the RNA N^6^-methyladenosine (m^6^A) modification patterns are extensively involved in the development of multiple tumors. However, the association between the m^6^A regulator expression patterns and the sarcoma tumor immune microenvironment (TIME) remains unclear.

Methods: We systematically evaluated the m^6^A regulator expression patterns in patients with sarcoma based on known 23 m^6^A regulators. Different m^6^A regulator expression patterns were analyzed using gene set variation analysis and a single-sample gene set enrichment analysis algorithm. According to the results of consensus clustering, we classified the patients into four different clusters. Next, we subjected the four clusters to differential genetic analysis and established m^6^A-related differentially expressed genes (DEGs). We then calculated the m^6^A-related DEGs score and constructed the m^6^A-related gene signature, named m^6^A score. Finally, the 259 sarcoma samples were divided into high- and low-m^6^A score groups. We further evaluated the TIME landscape between the high- and low-m^6^A score groups.

Results: We identified four different m^6^A modification clusters and found that each cluster had unique metabolic and immunological characteristics. Based on the 19 prognosis-related DEGs, we calculated the principal component analysis scores for each patient with sarcoma and classified them into high- and low-m^6^A score groups.

Conclusions: The m^6^A regulator expression patterns and complexity of the sarcoma TIME landscape are closely related to each other. Systematic evaluation of m^6^A regulator expression patterns and m^6^A scores in patients with sarcoma will enhance our understanding of TIME characteristics.

## INTRODUCTION

RNA modification is a form of post-transcriptional regulation, and RNA methylation accounts for 60% of all RNA modifications, among which the N^6^-methyladenosine (m^6^A) methylation is the most common type [1, 2]. The m^6^A methylation of RNA is a methylation modification formed by the 6th N of adenine (A) catalyzed by methyltransferase [3]. All m^6^A methylation reactions require the involvement of methyltransferases (Writers), demethylases (Erasers), and m^6^A-binding proteins (Readers) to perform the biological functions [4]. As a dynamic and reversible modification, m^6^A modification can directly or indirectly affect the biological processes of RNA transport, degradation, translation, and splicing [1]. Increasing evidence suggests that disordered expression of m^6^A regulators is closely associated with tumor immunity, tumor microenvironment, tumorigenesis, and tumor metastasis [5–7].

Sarcoma is a group of highly heterogeneous tumors that originate from the mesenchymal stromal cells. The five-year survival rate is 10–20% in patients with advanced sarcoma [8]. In clinical practice, the lack of knowledge of the molecular mechanisms of sarcoma development often leads to limitations in treatment tools and delays in the treatment period, especially in patients with advanced sarcomas. At present, the molecular regulatory mechanisms of sarcoma have not yet been fully elucidated. Notably, an increasing number of studies have shown that changes in the tumor immune microenvironment (TIME) play a key role in the occurrence and development of sarcoma [9, 10].

TIME is a complex ecosystem containing adaptive and innate immune cells with both pro-and anti-tumor properties [11]. Due to the diversity of its composition, TIME has a variety of possible cancer treatment targets [12]. With the development of immunotherapy, the treatment of malignancies has changed dramatically. Immunotherapy with immunological checkpoint inhibitors (ICIs), cytotoxic T lymphocyte-associated antigen-4 (CTLA-4), programmed cell death protein-1 (PD-1), and PD-ligand 1 (PD-L1) have demonstrated impressive clinical efficacy in patients with sarcoma [13, 14]. Unfortunately, 77.1% of patients with sarcoma receive little or no clinical benefit from ICIs [15]. Thus, TIME can identify different tumor immunophenotypes and improve the ability to guide and predict the immunotherapy response by comprehensively resolving the heterogeneity and complexity of immune cells in TIME [16].

Recent studies have revealed a potential connection between TIME and m^6^A modifications in various types of cancers [17, 18]. In a study of gastric cancer, Bo Zhang et al. demonstrated that assessment of m^6^A regulator expression patterns could predict the inflammation level, subtype, genetic variation, patient prognosis, and TIME [7]. Xin Liu et al. showed that methyltransferase-like (METTL)-14 (an m^6^A writer) overexpression inhibited gastric cancer cell proliferation and invasion by regulating the phosphoinositide 3-kinase/serine-threonine kinase/mammalian target of rapamycin (PI3K/AKT/mTOR) signaling pathway [19]. In addition, Botai et al. demonstrated that circNDUFB2 (a circular RNA) is involved in the degradation of the insulin-like growth factor 2 (IGF2) mRNA-binding protein 2 (IGF2BP2) (an m^6^A reader) during the progression of non-small cell lung cancer, which in turn activates anti-tumor immunity [20]. However, the potential roles of m^6^A modifications in the TIME of sarcomas remain unclear. Understanding the relationship between m^6^A methylation and TIME is important for further understanding the changes in immune transition during the development of sarcoma and accurately identifying potential new targets for the early diagnosis and effective treatment of sarcoma.

Therefore, a systematic evaluation of the connection between m^6^A regulator expression patterns and TIME will help improve our understanding of the sarcoma immune transition. In this study, we integrated genomic information from 259 sarcoma samples to systematically analyze m^6^A regulator expression patterns and combine them with the TIME landscape. We identified four m^6^A modification clusters and established an m^6^A scoring system to quantify individual patients with sarcoma to predict and guide their immunotherapy.

## RESULTS

### Landscape of m^6^A regulators in sarcoma

**Figure 1:**
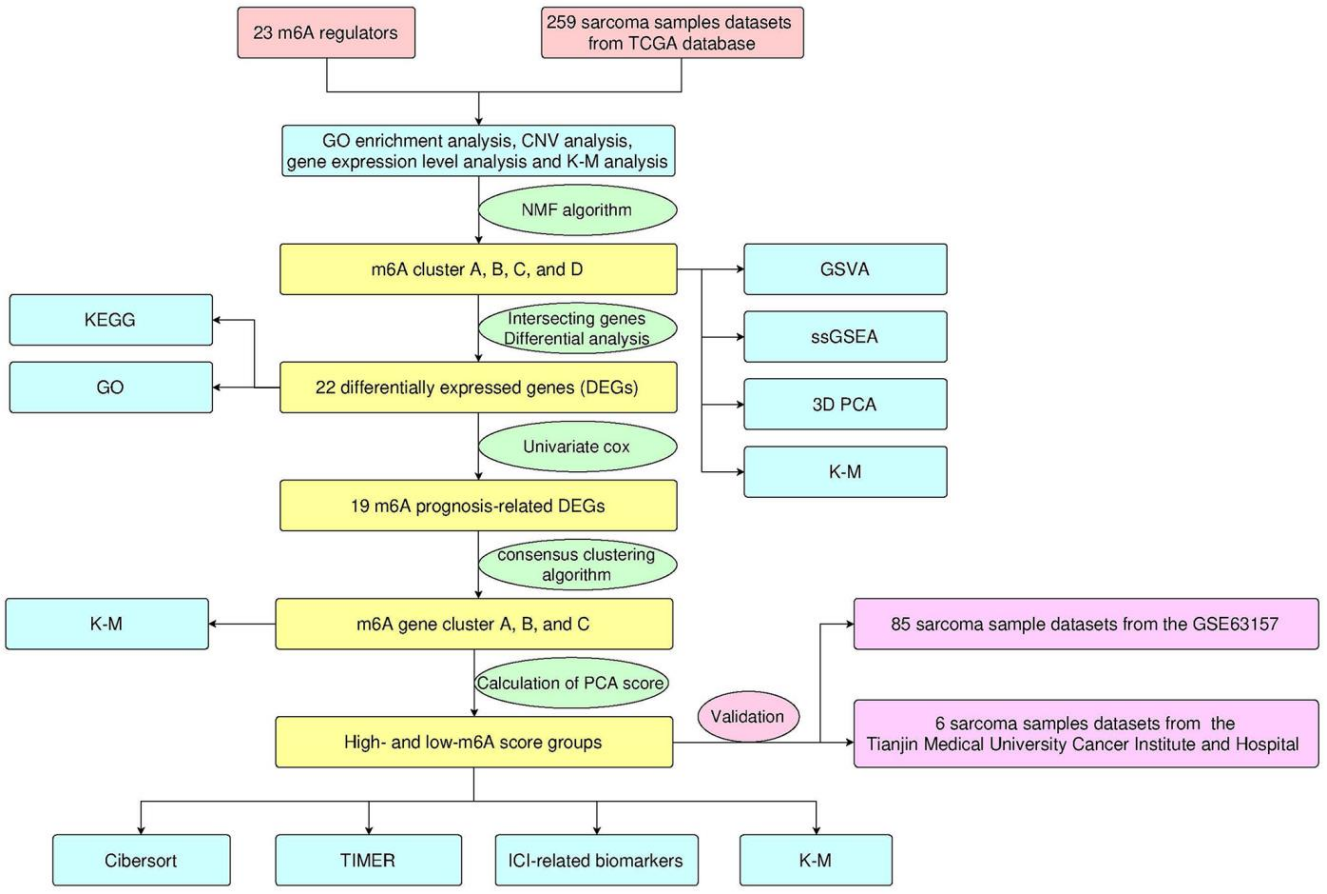
Design and workflow of the study.

**Figure 2:**
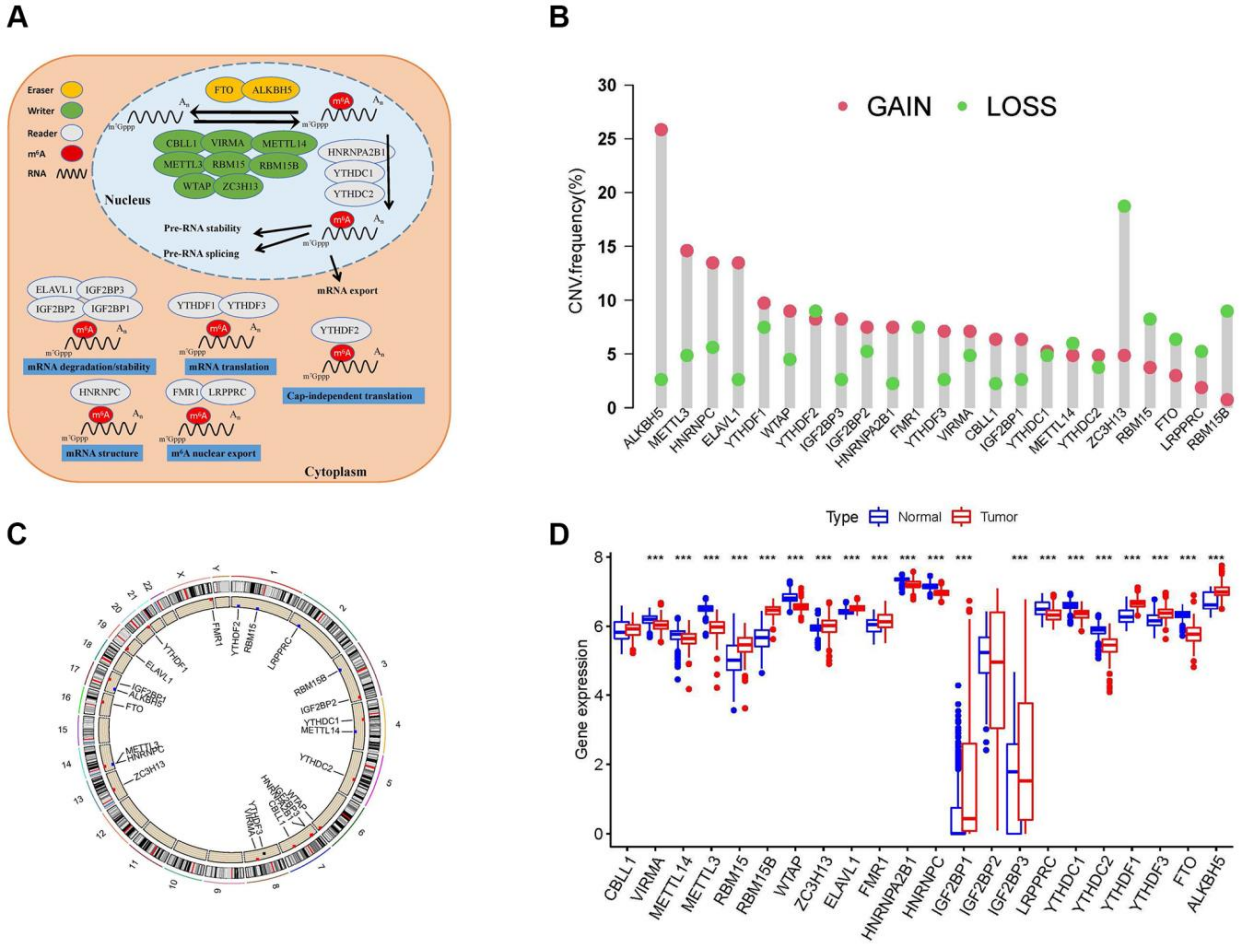
**Landscape of N^6^-methyladenosine (m^6^A) regulators in patients with sarcoma.** (**A**) Regulation of m^6^A regulators and their biological functions in RNA metabolism. A total of 23 known m^6^A regulators, including 13 readers, 8 writers, and 2 erasers. m^6^A methylation involves multiple stages of the RNA life cycle, including pre-mRNA shearing, pre-mRNA splicing, pre-mRNA transport, RNA translation, RNA degradation, etc. (**B**) Copy number variation (CNV) mutations in 23 m^6^A regulators. (**C**) The location of CNV alterations of m^6^A regulators on chromosomes. (**D**) Differential expression levels of 23 m^6^A regulators between normal tissues (Adipose and muscle tissues) and tumor tissues (sarcoma samples). Asterisks represent the statistical *P*-values (^*^P < 0.05; ^**^P < 0.01; ^***^P < 0.001).

The workflow of our study is shown in Figure 1. Through literature search and analysis, we identified a total of 23 m^6^A regulators, including 13 readers, eight writers, and two erasers (Supplementary Table 1). We summarized the process by which m^6^A regulators could add or remove m^6^A modification sites and change the potential biological functions of RNA splicing, translation, and degradation (Figure 2A). The investigation of copy number variation (CNV) alteration frequency showed a prevalent CNV alteration in 23 m^6^A regulators, and ALKB homolog 5 (ALKBH5), METTL3, heterogeneous nuclear ribonucleoprotein (HNRNP)-C, and ELAV-like 1 (ELAVL1) showed widespread CNV frequency gains. In contrast, METTL15, zinc finger CCCH-type containing 13 (ZC3H13), fat mass and obesity-associated (FTO), leucine-rich pentatricopeptide repeat containing (LRPPRC), and RNA-binding motif protein 15B (RBM15B) showed widespread CNV frequency loss (Figure 2B). We further searched for the position of CNV alteration in the m^6^A regulator on the chromosome (Figure 2C). We can completely distinguish sarcoma samples (259 sarcoma samples from The Cancer Genome Atlas [TCGA] database) from normal samples (911 muscle and adipose tissue samples from the University of California Santa Cruz [UCSC] Xena database) based on the expression levels of these 23 m^6^A regulators (Figure 2D). The median overall survival (OS) of patients with sarcoma was found to be 2.57 years (IQR = 1.3–4.34 years). Among them, Vir-Like m^6^A methyltransferase associated (VIRMA), METTL14, METTL3, Wilms tumor 1 (WT1)-associated protein (WTAP), fragile-X mental retardation 1 (FMR1), HNRNPA2B1, HNRNPC, IGF2BP3, LRPPRC, YTH domain containing (YTHDC)-1, YTHDC2, and FTO were highly expressed in normal tissues, while RBM15, RBM15B, ZC3H13, ELAVL1, IGF2BP1, YTH m^6^A RNA-binding protein (YTHDF)-1, YTHDF3, and ALKBH5 were highly expressed in sarcoma tissues. The expression levels of Cbl-like 1 (CBLL1) and IGF2BP2 in normal and sarcoma tissues were not statistically significant. Gene Ontology (GO) enrichment results are shown in Supplementary Figure 1A. We then used Cox regression analysis and Kaplan-Meier (K-M) analysis to determine the relationship between m^6^A regulators and the prognosis of patients with sarcoma. Univariate Cox regression analysis showed that the expression levels of IGFBP2 (*P*-value = 0.002), VIRMA (*P*-value = 0.039), IGFBP1 (*P*-value <0.001), HNRNPC (*P*-value = 0.009), HNRNPA2B1 (*P*-value = 0.004), YTHDF2 (*P*-value <0.001), and IGF2BP3 (*P*-value = 0.005) were protective factors for patients with sarcoma (Supplementary Figure 1B and Supplementary Table 2). At the same time, we calculated the K-M value of m^6^A regulators in patients with sarcoma by each best cut-off point (Supplementary Table 3) and the survival curve of m^6^A regulators (Supplementary Figure 2A–2W).

### Identification of m^6^A methylation modification patterns

**Figure 3:**
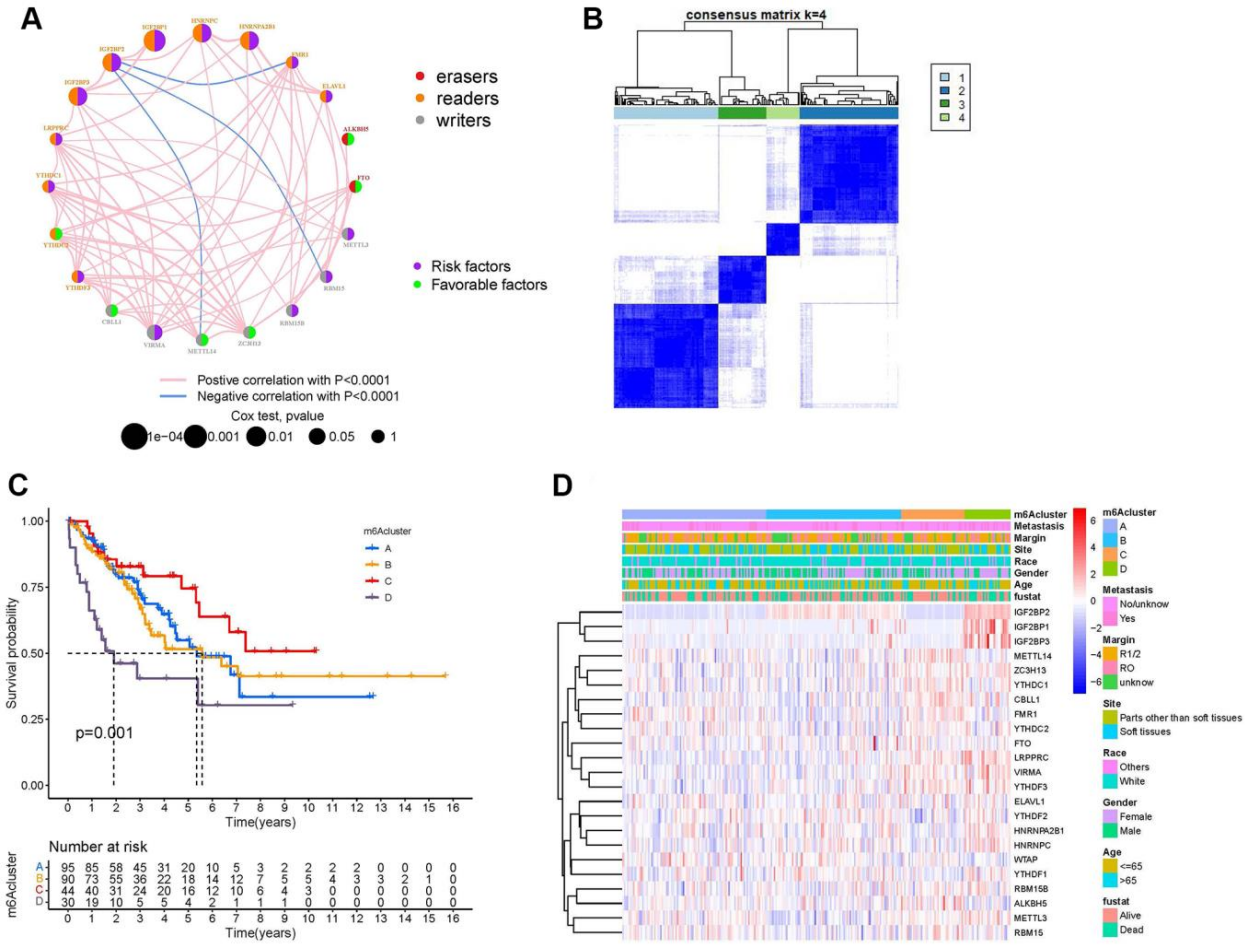
**Establishment of m^6^A methylation modification patterns.** (**A**) Regulatory functions and interactions of the 23 m^6^A regulators in sarcoma. The size of the circle represents the influence of different regulators on prognosis, while the Log-rank test was used to calculate values ranging from *P* < 1e^−4^, *P* < 0.001, *P* < 0.01, *P* < 0.05, and *P* > 0.05. Purple dots in circles, prognostic risk factors; green dots in circles, prognostic favorable factors. Erasers, readers, and writers are indicated by red, orange, and gray dots, respectively. The regulators' lines show their interactions, with negative correlations marked in blue and positive correlations in red. (**B**) The consensus matrix heatmap defined four m^6^A methylation modification clusters from 259 patients with sarcoma. (**C**) Kaplan-Meier curves of the overall survival rates of 259 patients with sarcoma with four m^6^A methylation modification clusters. (**D**) The heat map shows the expression levels of 23 m^6^A regulators in different m^6^A modification clusters and various clinicopathological features.

The m^6^A regulator network describes the association between the 23 m^6^A regulators, their interactions, and their prognostic significance in patients with sarcoma (Figure 3A). The results of the m^6^A regulatory network indicate that the interaction between writers, readers, and erasers may play unique roles in the formation of different m^6^A regulator expression patterns and affect the prognosis of patients with sarcoma [21, 22]. We used non-negative matrix factorization (NMF) consistent clustering to classify the sarcoma samples and identified four m^6^A modification clusters (Figure 3B and Supplementary Figure 3A–3J), namely, m^6^A-cluster-A, m^6^A-cluster-B, m^6^A-cluster-C, and m^6^A-cluster-D. K-M analysis showed that m^6^A-cluster-C had a significant survival advantage, while m^6^A-cluster-D showed a poor prognosis (Figure 3C). A heat map showed the differential expression patterns of 23 m^6^A regulators in four different m^6^A modification clusters (Figure 3D).

### TIME landscape characteristics in four m^6^A regulator expression patterns

**Figure 4:**
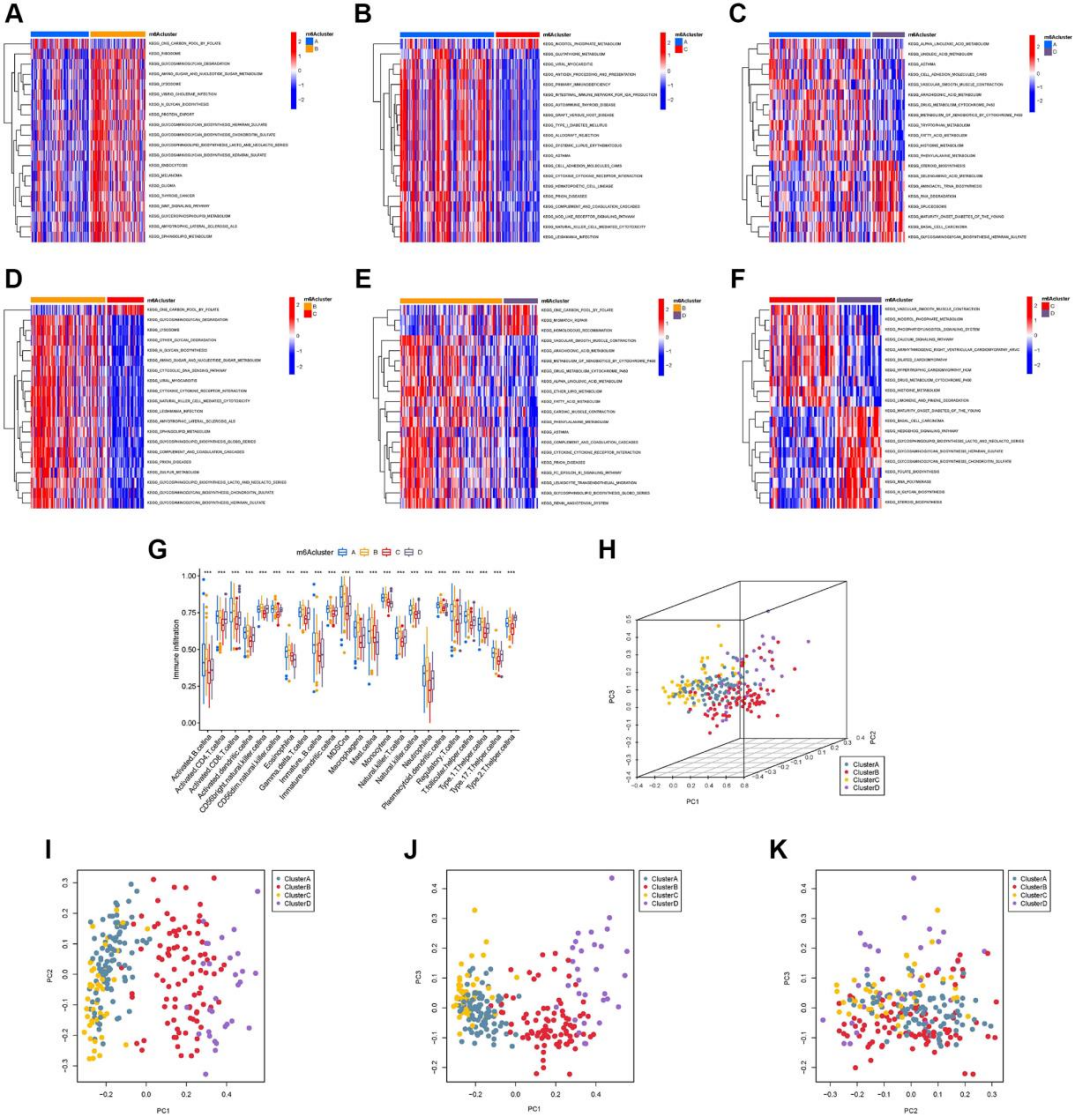
**Gene set variation analysis (GSVA), single sample gene set enrichment analysis, and principal component analysis (PCA).** The heat map shows the GSVA scores of representative hallmark passages by comparing two by two m^6^A regulator expression patterns. (**A**) m^6^A cluster-A vs. m^6^A cluster-B; (**B**) m^6^A cluster-A vs. m^6^A cluster-C; (**C**) m^6^A cluster-A vs. m^6^A cluster-D; (**D**) m^6^A cluster-B vs. m^6^A cluster-C; (**E**) m^6^A cluster-B vs. m^6^A cluster-D; (**F**) m^6^A cluster-C vs. m^6^A cluster-D; (**G**) Abundance of infiltrating cells in each of the four m^6^A modification patterns. Asterisks represent the statistical *P*-values (^*^P < 0.05; ^**^P < 0.01; ^***^P < 0.001). (**H**) Three-dimensional PCA (3D PCA) results of four m^6^A modification clusters, showing significant differences in the transcriptome between different modification clusters. (**I**) Projection of 3D PCA on the first principal component (PC1) and the second principal component (PC2). (**J**) Projection of 3D PCA on PC1 and the third principal component (PC3). (**K**) Projection of 3D PCA on PC2 and PC3.

To explore the biological behavior of the four different m^6^A modification types, we performed pairwise gene set variation analysis (GSVA) enrichment analysis (Figure 4A–4F). GSVA results showed that m^6^A-cluster-A was significantly rich in material metabolism (amino acid and lipid metabolism) and human diseases (immune-related, such as systemic lupus erythematosus and primary immunodeficiency). It is worth noting that material metabolism-related processes, especially glucose metabolism, are enriched in m^6^A-cluster-B. The pathways enriched by m^6^A-cluster-C are phosphoinositide transduction and metabolism and human diseases (circulatory system related). The pathways enriched by m^6^A-cluster-D are mainly metabolism-related pathways (such as glycan metabolism and lipid metabolism) and human disease-related signaling pathways (basal cell carcinoma and diabetes). Surprisingly, GSVA results showed that the signaling pathways with high expression between different clusters were mostly material metabolism- and immune-related, further confirming a direct or indirect link between m^6^A regulator expression patterns and TIME. In addition, we constructed a visible box-line plot by the “ssGSEA” package to compare the differences in the relative abundance of 28 immune infiltrating cell subpopulations in 4 different m^6^A clusters (Figure 4G). Statistically significant differences in immune cell infiltration results indicate the reliability of the m^6^A cluster in elucidating the immunophenotyping of TIME. Three-dimensional PCA (3D PCA) analysis was performed in four different m^6^A clusters based on the expression of 23 m^6^A regulators. 3D PCA and its projection on the three planes (Figure 4H–4K) showed that the distribution of four different m^6^A modification clusters was disorderly.

### Generation of m^6^A gene signatures and functional annotation

**Figure 5:**
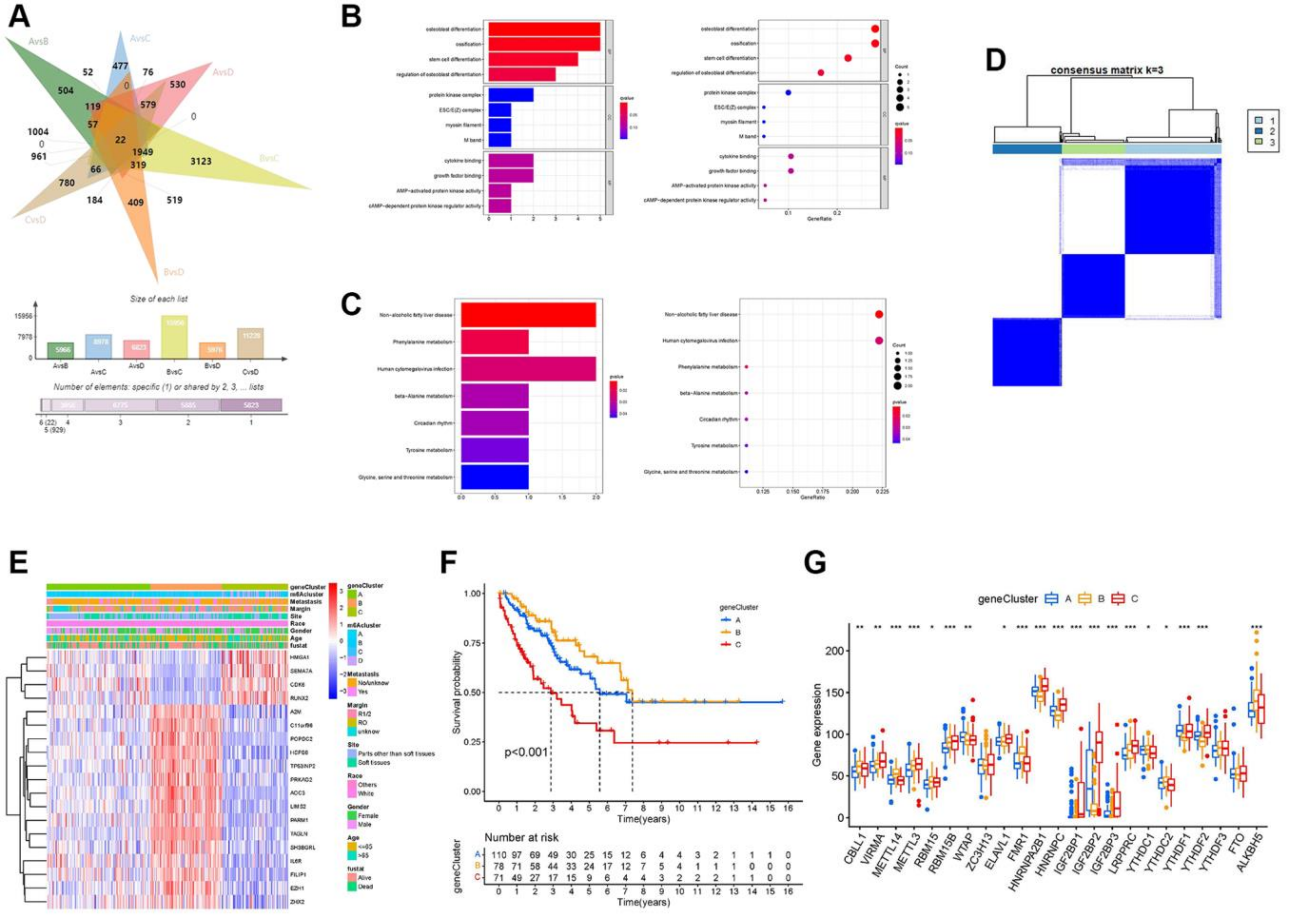
**Development of the m^6^A score and exploration of functional annotation.** (**A**) Venn diagram showing 22 m^6^A-associated differentially expressed genes (DEGs) between the six clusters compared. (**B**) Functional annotation of m^6^A-related DEGs using Gene Ontology (GO) enrichment analysis. (**C**) Functional annotation of m^6^A-related DEGs using Kyoto Encyclopedia of Genes and Genomes (KEGG) enrichment analysis. (**D**) The consensus matrix heatmap defined three m^6^A gene clusters. (**E**) The heat map shows the expression levels of 22 m^6^A-related DEGs in different m^6^A gene clusters and various clinicopathological features. (**F**) Kaplan-Meier curves of the overall survival rates of 259 patients with sarcoma with three m^6^A gene clusters. (**G**) Expression levels of 23 m^6^A regulators in each of the three m^6^A gene clusters. Asterisks represent the statistical *P*-values (^*^P < 0.05; ^**^P < 0.01; ^***^P < 0.001).

To deeply explore the potential biological functions of the four m^6^A regulator expression patterns, we analyzed the differentially expressed genes (DEGs) of the two m^6^A modification clusters using the “limma” package and took the intersection (Figure 5A and Supplementary Table 4). Finally, we identified 22 m^6^A-related DEGs (Supplementary Table 5). Surprisingly, based on the results of GO (Figure 5B) and Kyoto Encyclopedia of Genes and Genomes (KEGG) (Figure 5C), these 22 m^6^A DEGs showed that tumor metabolism was closely related to m^6^A modification, which reinforces that m^6^A modification plays a non-negligible role in TME. We further performed univariate Cox analysis on 22 DEGs and obtained 19 prognosis-related DEGs (Supplementary Table 6). To further validate the mechanism between m^6^A and TIME, we performed consensus clustering analysis based on the 19 m^6^A prognosis-related DEGs. We classified the samples into three different genomic subtypes and named these three clusters as m^6^A gene clusters A, B, and C (Figure 5D and Supplementary Figure 4A–4J). Heat map showing differences in the expression of 19 m^6^A prognosis-related DEGs in the three m^6^A gene clusters (Figure 5E). K-M analysis results showed that m^6^A gene cluster B had a significant survival advantage, while m^6^A gene cluster C had a poor prognosis (Figure 5F). Immediately afterward, we found significant differences in the expression of m^6^A regulators among the three m^6^A gene clusters (Figure 5G).

### Development of the m^6^A score and its clinical significance

**Figure 6:**
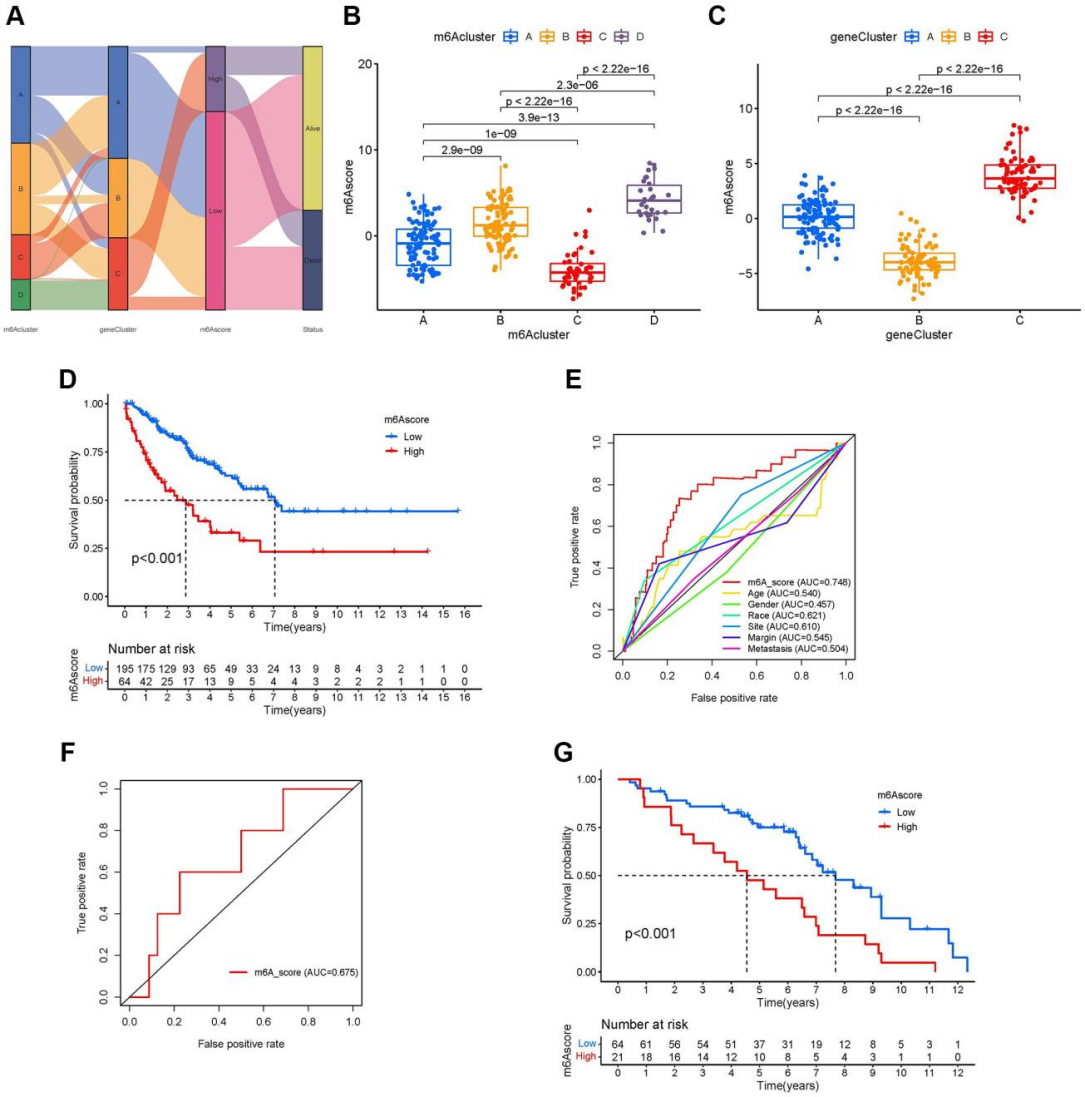
**Development of the m^6^A score and exploration of m^6^A-related clinical features.** (**A**) Sankey diagrams of different m^6^A modification clusters, m^6^A gene clusters, m^6^A scores, and different prognosis status. (**B**) Differences in the m^6^A scores among four m^6^A modification clusters (*P*-value <0.001, Kruskal-Wallis test). (**C**) Differences in the m^6^A scores among three m^6^A gene clusters (*P*-value <0.001, Kruskal-Wallis test). (**D**) Survival analyses for low-and high-m^6^A score groups using Kaplan-Meier curves (*P*-value < 0.001, Log-rank test). (**E**) Comparison of the area under the receiver operating characteristic curve between the m^6^A score and clinicopathological characteristics. (**F**) The receiver operating characteristic curve of m^6^A score in the validation set GSE63157 (Area under curve = 0.675). (**G**) Survival analyses for low- and high-m^6^A score groups using Kaplan-Meier curves in validation set GSE63157 (*P*-value < 0.001, Log-rank test).

Although our studies have further confirmed the relationship between m^6^A modification in TIME and prognosis, these analyses cannot predict the pattern of m^6^A modification in patients with sarcoma and cannot assess the prognosis of these patients. Therefore, we developed a scoring scheme called the m^6^A score to quantify the m^6^A regulator expression pattern in each sarcoma patient (Supplementary Table 7). We used a Sankey plot to illustrate the workflow (Figure 6A). The Kruskal-Wallis test showed that there were significant differences in m^6^A scores between the m^6^A clusters. The median of m^6^A cluster C was the lowest, and the median of m^6^A cluster D was the highest, indicating that a high m^6^A score may be closely related to the characteristics of material metabolism, while a low m^6^A score may be related to immune cell infiltration (Figure 6B). At the same time, m^6^A gene cluster C showed the highest median score of m^6^A, and m^6^A cluster B showed the lowest median score (Figure 6C). The results of the K-M curve showed that patients with high m^6^A scores had a shorter survival time than those with a low score group (Figure 6D). In different clinicopathological characteristics (age, race, sex, site, margin status, and metastasis status), it was further confirmed that the prognosis of patients with high m^6^A scores was worse than that of patients with low m^6^A scores (Supplementary Figure 5A–5L). We drew the receiver operating characteristic (ROC) curve to further explore the prediction function of the m^6^A score for patients with sarcoma. We found that the AUC of m^6^A score was 0.748, which is much larger than the AUC of other clinicopathological features, and the m^6^A score can predict the prognosis of patients with sarcoma (Figure 6E). The m^6^A score was validated in GSE63157, and the AUC value of the validation cohort was 0.675 (Figure 6F). Additionally, the validation cohort GSE63157, survival curves showed that low-risk patients had a favorable prognosis than high-risk patients (*P* < 0.001, Figure 6G).

### Role of the m^6^A score in immunotherapy

**Figure 7:**
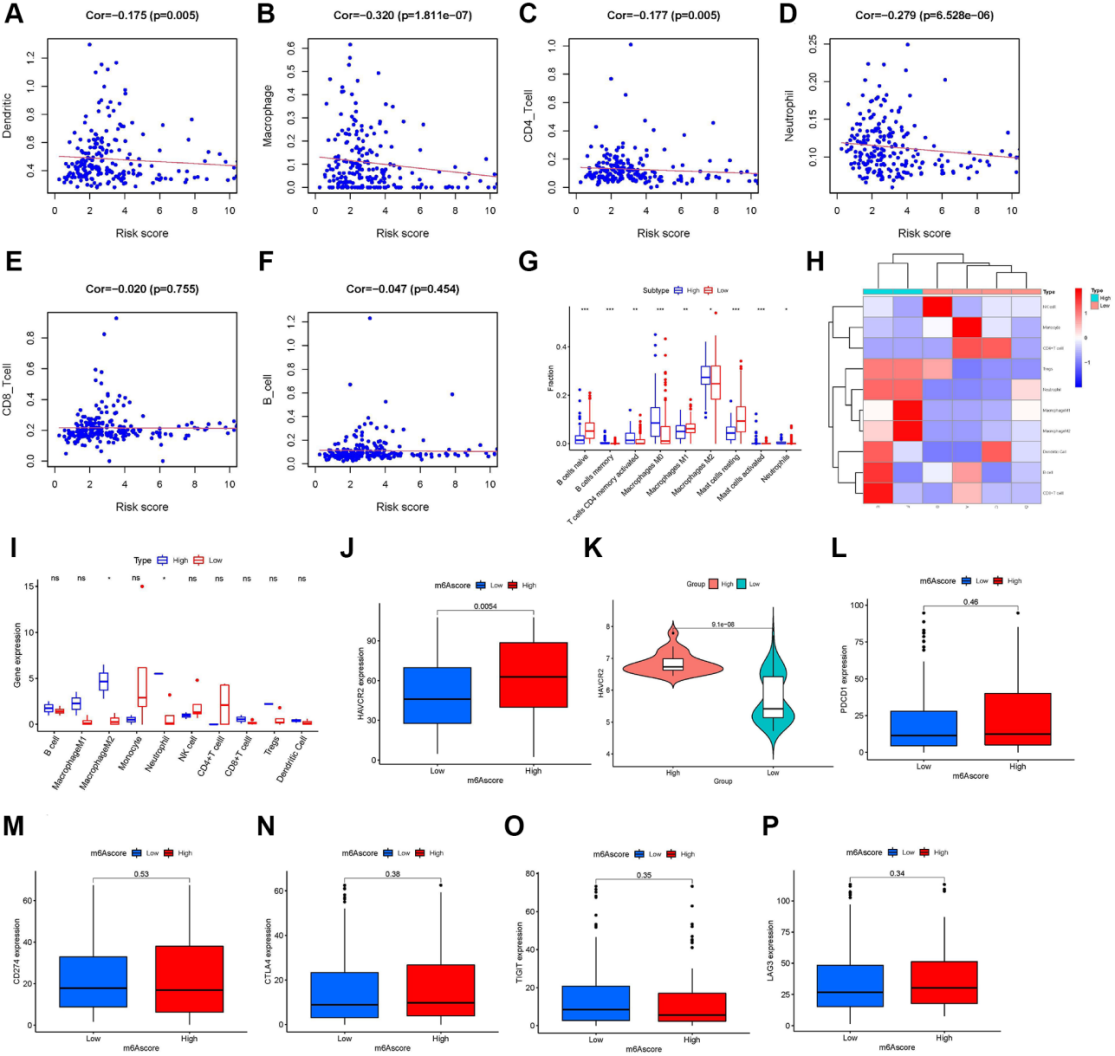
**Role of the m^6^A score in immunotherapy.** Infiltration abundances of six types of immune cells (Pearson correlation analysis). (**A**) Dendritic Cells (Cor = –0.175, *P*-value = 0.005). (**B**) Macrophages (Cor = –0.320, *P*-value <0.001). (**C**) CD4^+^ T cells (Cor = –0.177, *P*-value = 0.005). (**D**) Neutrophil (Cor = –0.279, *P*-value <0.001). (**E**) CD8^+^ T cells (Cor = –0.020, *P*-value = 0.755). and (**F**) B cells (Cor = –0.047, *P*-value = 0.454). (**G**) Box plots visualizing significantly different immune cells between high- and low-m^6^A score groups. Infiltration degrees of naive B cells, M1 macrophages, and resting mast cells were higher in the low-m^6^A score group than the high-m^6^A score group. Meanwhile, the infiltration degrees of memory B cells, activated memory CD4 T cells, M0 and M2 macrophages, and activated mast cells were higher in the high-m^6^A score group than the low-m^6^A score group. (**H**) Ten immune cells infiltration heat map of the six patients from Tianjin Medical University Cancer Institute and Hospital. (**I**) Infiltration degrees of M2 macrophages and neutrophils were higher in the high-m^6^A score group than the low-m^6^A score group in the six patients validation set. The box plot shows the differences in (**J**) hepatitis A virus cellular receptor 2 (HAVCR2) between high- and low-m^6^A score groups in TCGA database, (**K**) HAVCR2 between high- and low-m^6^A score groups in the validation set, GSE63157, and (**L**) programmed cell death 1 (PD-1), (**M**) CD274, (**N**) cytotoxic T lymphocyte-associated antigen-4 (CTLA-4), (**O**) T cell immunoglobulin and ITIM domain (TIGIT), and (**P**) lymphocyte activation gene-3 (LAG3) between high- and low-m^6^A score groups in The Cancer Genome Atlas (TCGA) database. Asterisks represent the statistical *P*-values (^*^P-value <0.05; ^**^P-value <0.01; ^***^P-value <0.001).

Using the Tumor Immune Estimation Resource (TIMER), we evaluated the association between the prognostic features and immune cell infiltration. The results indicated that DC (Cor = –0.175, *P*-value = 0.005, Figure 7A), macrophages (Cor = –0.320, *P*-value <0.001, Figure 7B), CD4^+^ T cells (Cor = –0.177, *P*-value = 0.005, Figure 7C), and neutrophils (Cor = −0.279, *P*-value <0.001, Figure 7D) were significantly negatively correlated with the m^6^A score. The other two types of immune cells, CD8^+^ T cells (Figure 7E) and B cells (Figure 7F) were not significantly different from the m^6^A score. Immune cell infiltration analysis results of TIMER further confirmed that a low m^6^A score might be related to immune cell infiltration. In addition, using CIBERSORT, we assessed the difference in immune cell infiltration between the high-m^6^A score group and the low-m^6^A score group. The results of CIBERSORT showed that the infiltration degrees of activated B cells, memory T cells, CD4^+^ T cells, M0 and M2 macrophages, and mast cells were higher in the high-m^6^A score group than in the low-m^6^A score group; in contrast, the infiltration degrees of naïve B cells, M1 macrophages, and resting mast cells were higher in the low-m^6^A score group than in the high-m^6^A score group (Figure 7G). Immune cell infiltration analysis results of CIBERSORT showed that different m^6^A scores had different immune cell types infiltrated in TIME. Based on the m^6^A score, we divided six patients from Tianjin Medical University Cancer Institute and Hospital into a low-m^6^A score group (four samples) and a high-m^6^A score group (two samples) and plotted their immune cell infiltration heat map (Figure 7H). By analyzing the degree of immune cell infiltration in the external validation set of the six patients, we further confirmed the conclusion that macrophage M2 and neutrophils were higher in the high-m^6^A score group than in the low-m^6^A score group (Figure 7I). The results of ICI-related biomarker analysis showed that the expression of TIM-3 (*p* = 0.005) was higher in the high-m^6^A score group than in the low-m^6^A score group (Figure 7J). The validation set GSE63157 high-m^6^A score group also had a higher level of TIM-3 expression than the low-m^6^A score group (Figure 7K). However, there was no statistical difference in the expression of PD1 (Figure 7L), PD-L1 (Figure 7M), CTLA4 (Figure 7N), T cell immunoglobulin and ITIM domain (TIGIT) (Figure 7O), and lymphocyte activation gene-3 (LAG3) (Figure 7P) between the high- and low-m^6^A score groups.

## DISCUSSION

With the development of next-generation sequencing technology, m^6^A modifications have been detected in innate immune diseases, inflammation, and various tumors [5, 6, 23]. m^6^A modifications can affect the transcriptional translation of mRNA and/or non-coding RNA of related genes by regulating the methylation at the cellular RNA level, thus activating cellular signal transduction pathways and affecting cell proliferation, differentiation, migration, invasion, apoptosis, DNA damage repair, etc. [24, 25]. Currently, most studies are focused on single or multiple m^6^A modulators, and there is a lack of comprehensive analysis of the role of m^6^A modulators in sarcoma TIME [22]. Recognition of the characteristics of different m^6^A regulator expression patterns in TIME will help enhance our understanding of TIME and allow for a more effective evaluation of patient prognosis and guidance of immunotherapy.

This study identified four different m^6^A clusters with different TIME characteristics, including different immune-related pathways, degree of immune cell infiltration, and different material metabolism-related pathways. Among the four different m^6^A clusters, m^6^A-cluster-C had a significant survival advantage, while m^6^A-cluster-D had a poor prognosis. This may be closely related to the high synthesis and metabolism of lipids and glycans in the TIME of m^6^A-cluster-D [26]. Jiang et al. demonstrated that increased glycolysis is closely associated with elevated immune activity in TIME [27]. In our study, this pattern may also exist in m^6^A-cluster-B, where both glucose metabolism and the level of immune cell infiltration were higher than other three m^6^A-clusters. Combining the immune cell infiltration characteristics of each m^6^A cluster with the PCA results, we further confirmed the reliability of the fact that different m^6^A regulator expression patterns have different immune phenotypes. Therefore, in this study, mRNA and/or non-coding RNA differences between different m^6^A clusters were significantly associated with m^6^A modification and TIME. However, the relationship between DEGs and TIME of different m^6^A clusters is unclear. Therefore, we searched for intersecting genes to further explore the relationship between m^6^A-related genes and TIME. We then used univariate Cox regression to analyze the intersection genes to identify 19 prognosis-related DEGs. These DEGs were considered m^6^A-related signature genes, and these DEGs were also closely associated with the TME. Interestingly, we used consensus clustering similar to m^6^A regulator expression patterns, and three m^6^A gene clusters were identified based on prognosis-related DEGs. The expression of 23 m^6^A regulators was statistically different among the three subtypes. Among them, m^6^A gene cluster B had the best prognosis, and m^6^A gene cluster C had the worst prognosis. These results again illustrate that m^6^A regulators are significantly associated with TIME. Thus, a comprehensive assessment of m^6^A-associated DEGs enhances our understanding of the TIME landscape with different m^6^A regulator expression patterns.

Given the need for individualized prediction and treatment, quantification of m^6^A regulator expression patterns in patients with sarcoma, and enhancing the understanding of the TIME landscape, there is an urgent need to develop a novel m^6^A scoring system. To this end, we have developed a novel scoring system to assess m^6^A regulator expression patterns in patients with sarcoma, named the m^6^A score. Based on the results of the m^6^A score, patients with sarcoma were divided into high- and low-m^6^A score groups based on the optimal cut-off point. Through the common results of the two analysis methods, we found that the degree of immune cell infiltration in patients with sarcoma with high m^6^A score group was lower than that in patients with low m^6^A score groups (e.g., dendritic cells, macrophages, CD4^+^ T cells, and neutrophils). It is not difficult to infer from the previous conclusion that the prognosis of patients with a high degree of immune cell infiltration is worse [28, 29].

Moreover, our results further confirmed that the higher the m^6^A score, the lower the degree of immune cell infiltration, and the worse the prognosis of patients with sarcoma. The expression of TIM-3 has been observed in various tumor cells and immune cells [30]. It has been well documented that both protein and mRNA expression levels of TIM-3 are elevated in tumor tissue samples and that elevated TIM-3 is associated with poor prognosis in various tumors, including sarcomas [31, 32]. In our study, we found that TIM-3 expression was higher in the high m^6^A score group than in the low m^6^A score group, and based on the findings of the existing studies [31, 32], we again confirmed that the prognosis of patients with sarcoma in the high m^6^A score group was worse than that in the low m^6^A score group. However, we did not find differential expression of other ICI-related immune markers (PD1, PD-L1, CTLA4, TIGIT, and LAG3) between the high and low m^6^A score groups.

To confirm the reliability of the conclusions of the analysis of sarcoma samples from TCGA database, we performed validation using two independent datasets. The validation set was GSE63157 from GEO and six patients from the Tianjin Medical University Cancer Institute and Hospital. The validation set of six patients may cause some problems, such as an extensive confidence interval range, due to the small sample size. However, the results reflected by the two validation sets combined confirmed the value of clinical use of the m^6^A score to predict survival and assess the TIME landscape of patients with sarcoma. The validation results confirm that the m^6^A score can improve our understanding of the TIME landscape of sarcomas and predict and guide the treatment of patients.

Although the intrinsic association between m^6^A regulators and TIME in patients with sarcoma has been preliminarily investigated through statistical and bioinformatic analyses, our study still has some shortcomings. First, although we reviewed the literature and compiled a catalog of 23 recognized RNA methylation regulators, more m^6^A regulators will be discovered over time with development. Therefore, novel defined m^6^A regulators need to be enrolled in the study to refine the accuracy of m^6^A regulator expression patterns and m^6^A scores. Second, the external validation set consisting of six samples leads to a large confidence interval because of the small sample size, and the improvement of its validation capability requires further expansion of the sample size. Third, m^6^A regulator expression patterns, m^6^A-related DEGs, and m^6^A scores were identified using retrospective datasets; therefore, *in vivo* and *in vitro* experiments and prospective cohorts of patients with sarcoma are needed to further validate our findings. Finally, patients with sarcoma with a poorer prognosis were also included in the low m^6^A score group; therefore, additional clinicopathologic features should be incorporated into the prediction model to improve accuracy.

## CONCLUSION

In this study, we comprehensively assessed the expression patterns of 23 m^6^A regulators in 259 sarcoma samples. m^6^A was evaluated integrally with TIME. This work further confirmed that m^6^A methylation modifications have important regulatory mechanisms in sarcoma TIMEs. Differences in m^6^A regulator expression patterns are responsible for the individual differences in sarcoma TIMEs. Therefore, a systematic evaluation of individual m^6^A regulator expression patterns will help to enhance our understanding of the sarcoma TIME landscape and predict and guide the treatment of affected patients.

## METHODS

### Data collection and processing

Gene expression datasets and corresponding clinical datasets (including age, race, gender, site, margin status, metastasis status, status, and survival months) for patients with sarcoma were downloaded from TCGA database (https://www.cancer.gov/). Patients with sarcomas without related overall survival were excluded. Gene expression datasets of normal muscle and adipose tissue samples were downloaded from the UCSC Xena database (https://xenabrowser.net/). To achieve comparability between gene expression data, we converted gene expression datasets of UCSC from Fragments Per Kilobase Million (FPKM) format to transcripts per kilobase million (TPM) format. Genome mutation data (CNV) of TCGA-SCAR were also downloaded from the UCSC database. By searching the literature related to m^6^A methylation modification, 23 confirmed m^6^A regulators were included in our study [7, 33, 34]. We downloaded the gene file of “c2.cp.kegg.v7.4.symbols.gmt” from the Molecular Signatures Database (MSigDB) for GSVA analysis. The 23 m^6^A regulators included 13 readers, 8 writers, and 2 erasers (Supplementary Table 1).

### Data collection for validation

We obtained gene expression profiles and clinical data for an independent cohort, GSE63157, from the Gene Expression Omnibus (GEO) database (https://www.ncbi.nlm.nih.gov/geo/). GSE63157 was used as an external validation cohort. We collected STS patients admitted to the Tianjin Medical University Cancer Institute and Hospital between 2016 and 2019. Tissue specimens from all patients were reviewed by pathologists and diagnosed with STS. All patients underwent whole-exome sequencing (WES). We quantified the scores of 10 immune cell types from the RNA-seq data of the six samples. The retrospective investigation was conducted in accordance with the Declaration of Helsinki and approved by the Ethics Committee of Tianjin Medical University Cancer Institute and Hospital (Approval No. E2019144). All patients provided signed informed consent. Genetic sequencing data were obtained from the tissue samples of six patients (the trial registration was NCT04126993) [35]. The Yuce Bio Company conducted gene sequencing procedures. Genomic DNA from formalin-fixed, paraffin-embedded (FFPE) sections from biopsy samples or whole blood control samples were extracted using the Gene Read DNA FFPE Kit (Qiagen, Germantown, MD, USA) and the Mag-Bind Blood and Tissue DNA HDQ 96 Kit (Qiagen), respectively. Library preparations were performed using the KAPA Library Quantification Kit (Roche, Indianapolis, IN, USA), and target enrichment was performed using the Target Seq Enrichment Kit (iGene Tech, Beijing, China), and sequencing was performed on a NovaSeq (Illumina, San Diego, CA, USA). The raw reads of WES-seq were processed using SOAPnuke (version 1.5.6, parameters: -l 20 -q 0.1 -n 0.1) to remove ambiguous reads and/or low-quality reads. These qualified sequence reads were then aligned to the human reference genome (UCSC hg38) using BWA-mem (BWA, version 0.7.12). We quantified the scores of 10 immune cell types from the RNA-seq data of the six samples.

### CNV analysis and differential analysis of m^6^A regulators and establishment of m^6^A regulator network

To explore the extent of mutation of 23 m^6^A regulators in sarcoma, we performed CNV analysis and labeled the positions of 23 m^6^A regulators in human chromosomes. Gene Ontology (GO) enrichment analysis based on 23 m^6^A regulators was performed. Combined with the datasets of sarcoma samples in TCGA and the datasets of muscle and adipose tissues in UCSC, we performed differential analysis. Immediately afterward, we combined the survival data of patients with sarcoma with K-M analysis for each m^6^A regulator and explored the interrelationships between the m^6^A regulators. Based on the results of the above analysis, an m^6^A regulator network was established.

### Consistent cluster analysis of 23 m^6^A regulators

NMF consensus clustering adds non-negative constraints to the decomposed matrix based on matrix factorization. For example, matrix A (M_A_) is decomposed into two non-negative matrices B (M_B_) and C (M_C_) (i.e., M_A_ ≈ M_B_ × M_C_, M_B_ ≥ 0, M_C_ ≥ 0) [36]. NMF consensus clustering was applied to identify different m^6^A regulator expression patterns by the “ConsensusClusterPlus” package, and we named the m^6^A regulator expression pattern the m^6^A cluster. The selection of the optimal number of clusters was determined by the cophenetic, consensus index, and silhouette coefficients. According to the consensus clustering results, we performed K-M analysis and plotted a heat map of patients in different m^6^A clusters.

### GSVA

GSVA is a non-parametric unsupervised analysis method that assesses whether different metabolic pathways are enriched among different clusters by combining the analysis of differences in gene expression between clusters [37]. We analyze the differences in biological processes between four different m^6^A regulator expression patterns by the “GSVA” package. Adjusted *P*-values less than 0.05, and log fold change (log FC) less than 0.1 were considered statistically significant.

### Estimation of immune cell infiltration and Principal Component Analysis (PCA)

The enrichment score calculated by single-sample gene set enrichment analysis (ssGSEA) represents the relative abundance of each immune cell in each m^6^A cluster [38]. PCA uses the idea of dimensionality reduction to transform K dimensions into M dimensions (K > M) with a small loss of information and uses M dimensions to explain the variance-covariance structure of multiple variables, thereby simplifying the system structure [39]. In our study, the N dimensions represent 23 m^6^A modulators, and the K dimensions represent two dimensions, which can be visualized. At the same time, we used different colors to distinguish the different m^6^A clusters.

### Identification of m^6^A related DEGs

To identify m^6^A-related genes, we divided patients into four different m^6^A clusters (Clusters A, B, C, and D) based on the expression of 23 m^6^A regulators. Next, we performed a two-by-two comparison of different clusters (Cluster AvsB, AvsC AvsD, BvsC, BvsD, and CvsD) to identify DEGs. We combined the DEGs intersection of the 6 groups (Cluster AvsB, AvsC AvsD, BvsC, BvsD, and CvsD) by jvenn [40]. Moreover, we obtained prognosis-related DEGs by performing univariate Cox regression analysis. The log FC and adjusted *P*-values were used to evaluate the significance of m^6^A DEGs. The filter criterion was set to log FC less than 2.00, and the adjusted *P*-value was less than 0.05.

### Establishment of m^6^A gene clusters and m^6^A score

We then established different m^6^A gene clusters to analyze the m^6^A DEGs. The process is as follows: (I) Analysis of differential genes using unsupervised clustering; (II) The number and stability of gene clusters were also defined by the consensus clustering algorithm. Based on the results of the m^6^A gene clusters, we performed a K-M analysis. Furthermore, to further quantify the m^6^A methylation modification in patients with sarcoma, we constructed a novel scoring system using prognosis-related m^6^A DEGs and named it the m^6^A score. The m^6^A score was constructed as follows: (I) We performed PCA and extracted the principal components 1 and 2 (PC1 and PC2); (II) Calculate the m^6^A score using the following equation:


$${\text{m}}^{6}\text{A score}=\sum ({\text{PC1}}_{i}+{\text{PC2}}_{i})$$

where *i* is the prognosis-related m^6^A DEGs.

Based on optimal cut-off points, patients with sarcoma were separated into high- and low m^6^A score groups. To further verify the m^6^A score, we used the Kruskal-Wallis test to evaluate whether the m^6^A score was different in the m^6^A cluster and the m^6^A gene cluster. To further demonstrate the characteristics of the m^6^A score, we assessed the survival status between high- and low-m^6^A score groups by K-M analysis. We also assessed survival status in different clinicopathological characteristics, including age (≤65, >65 years), race (white and others [Asian, black, and African American]), sex (female and male), site (soft tissues and parts other than soft tissues [uterus and retroperitoneum, etc.]), margin status (R0 and R1/2), and metastasis status (yes and no/unknown), according to high- and low-m^6^A score groups. To further explore the predictive function of the m^6^A score for patients with sarcoma, we drew the receiver operating characteristic curve (ROC) and calculated the area under the curve (AUC) for the m^6^A score and the different clinicopathological characteristics. To further test the accuracy of the m^6^A score, we calculated the m^6^A score values of the independent cohort (GSE63157) and plotted the ROC curve and K-M analysis.

### Correlation between m^6^A score and tumor immune microenvironment

First, we analyzed the differences in ICI-related biomarkers (CTLA-4, PD-1/PD-L1, hepatitis A virus cellular receptor 2/T cell immunoglobulin domain and mucin domain-3 [HAVCR2/TIM-3], lymphocyte activation gene 3 [LAG3], and T cell immunoreceptor with immunoglobulin and ITIM domain [TIGIT]) between the high- and low-m^6^A score groups. Second, we used the CIBERSORT database to identify a complex association between 22 different immune cells and different m^6^A score groups [41]. Third, TIMER [42] (https://cistrome.shinyapps.io/timer/) was used to systematically analyze and estimate the abundance of the six immune cell immune infiltrates in the m^6^A score. Finally, we used the set GSE63157 from GEO and six samples from Tianjin Medical University Cancer Institute and Hospital to verify the relationship between the m^6^A score and TIME.

### Statistical analyses

Statistical analyses in this study were performed using Ri386-4.0.3. Differences in quantitative data and normally distributed variables were compared using the *t*-test, and differences in non-normally distributed variables were compared using the Wilcoxon rank-sum test. Differences were compared for more than two groups of variables using one-way analysis of variance and the Kruskal-Wallis test. Prognostic analysis was performed using the Kaplan-Meier survival analysis and Cox proportional hazards model. Pearson’s analysis was used for the correlation analysis. A *P*-value <0.05 (two-tailed) was considered to indicate statistical significance. The Benjamini-Hochberg method was used to control for FDR for multiple hypothesis testing.

### Ethics approval and consent to participate

TCGA, UCSC, and GEO data did not involve animal experiments, human specimens, or ethics-related issues. Ethical approval for the six patients was obtained from the research ethics committee of the Cancer Institute and Hospital of Tianjin Medical University prior to the study. Written informed consent was obtained from all patients and/or their families. All specimens were handled and stored anonymously according to ethical and legal standards.

## Supplementary Materials

Supplementary Figures

Supplementary Tables 1-3

Supplementary Table 4

Supplementary Tables 5 and 6

Supplementary Table 7
