# Supplementary material for: m6A regulator-mediated methylation modification patterns and tumor immune microenvironment in sarcoma
Source: Aging (Albany NY). 2022 Jan 3;14(1):330–53. doi: 10.18632/aging.203807 (PMC8791212; doi:10.18632/aging.203807)
Supplement: Supplementary Figures [file aging-14-203807-s001.pdf]

SUPPLEMENTARY FIGURES

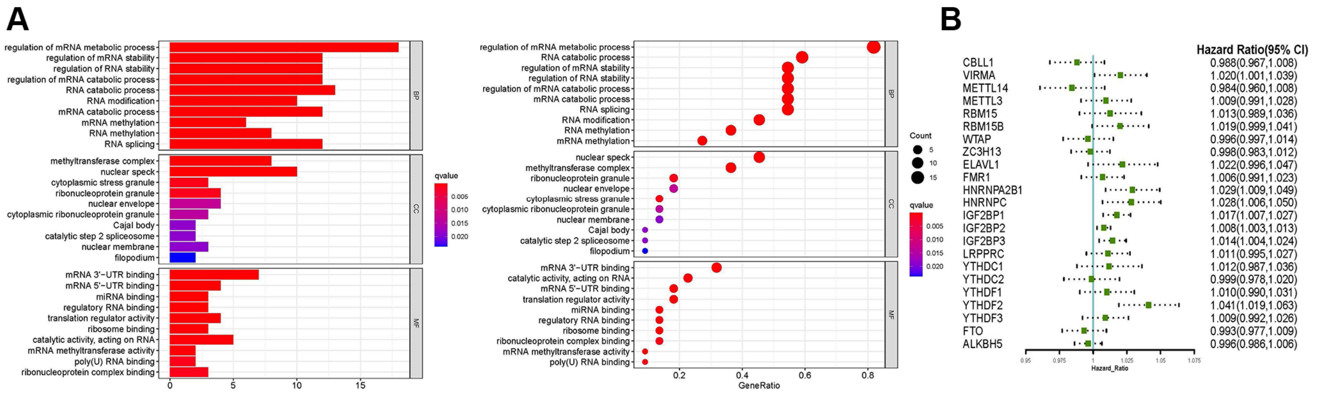

Supplementary Figure 1. The Gene Ontology (GO) enrichment and univariate Cox regression analyses results of 23 N^6-methyladenosine (m^6A) regulators. (**A**) GO enrichment of 23 m^6A regulators. (**B**) Univariate Cox regression analysis results of 23 m^6A regulators. Expression levels of insulin-like growth factor 2 (IGF2) mRNA-binding protein (IGFBP)-2 (*P*-value = 0.002), Vir-Like m^6A methyltransferase associated (VIRMA) (*P*-value = 0.039), IGFBP1 (*P*-value <0.001), heterogeneous nuclear ribonucleoprotein (HNRNP)-C (*P*-value = 0.009), HNRNPA2B1 (*P*-value = 0.004), YTH m^6A RNA-binding protein 2 (YTHDF2) (*P*-value <0.001). and IGF2BP3 (*P*-value = 0.005) were protective factors for patients with sarcoma.

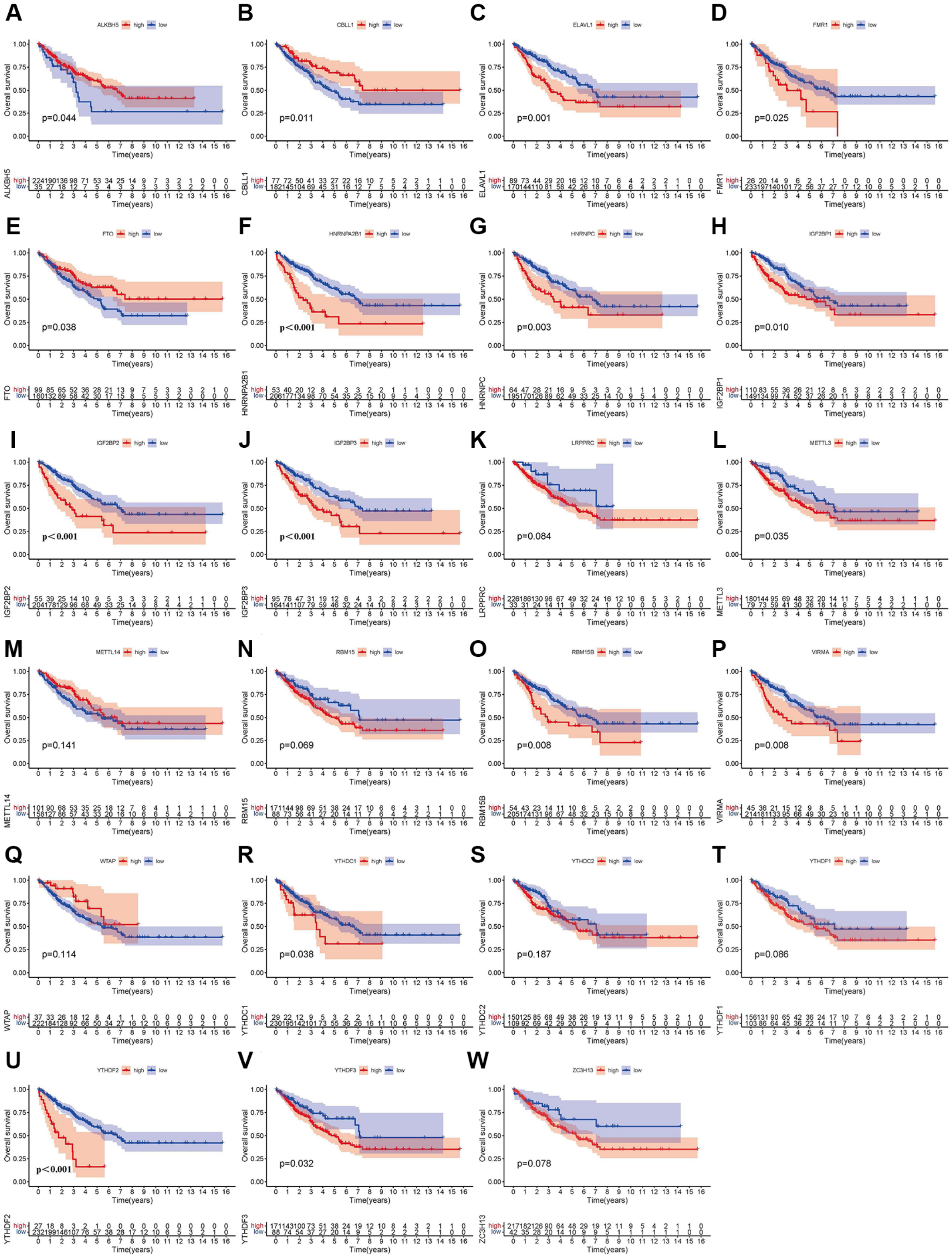

Supplementary Figure 2. Survival curve of 26 m^{6}A regulators. (**A**) ALKB homolog 5 (ALKBH5); (**B**) Cbl-like 1 (CBLL1); (**C**) ELAV-like 1 (ELAVL1); (**D**) Fragile-X mental retardation 1 (FMR1); (**E**) Fat mass and obesity-associated (FTO); (**F**) HNRNPA2B1; (**G**) HNRNPC; (**H**) IGF2BP1; (**I**) IGF2BP2; (**J**) IGF2BP3; (**K**) leucine-rich pentatricopeptide repeat containing (LRPPRC); (**L**) Methyltransferase-like (METTL)-3; (**M**) METTL14; (**N**) RNA-binding motif protein 15 (RBM15); (**O**) RBM15B; (**P**) VIRMA; (**Q**) Wilms tumor 1 (WT1)-associated protein (WTAP); (**R**) YTH domain containing (YTHDC)-1; (**S**) YTHDC2; (**T**) YTHDF1; (**U**) YTHDF2; (**V**) YTHDF3; (**W**) Zinc finger CCCH-type containing 13 (ZC3H13).

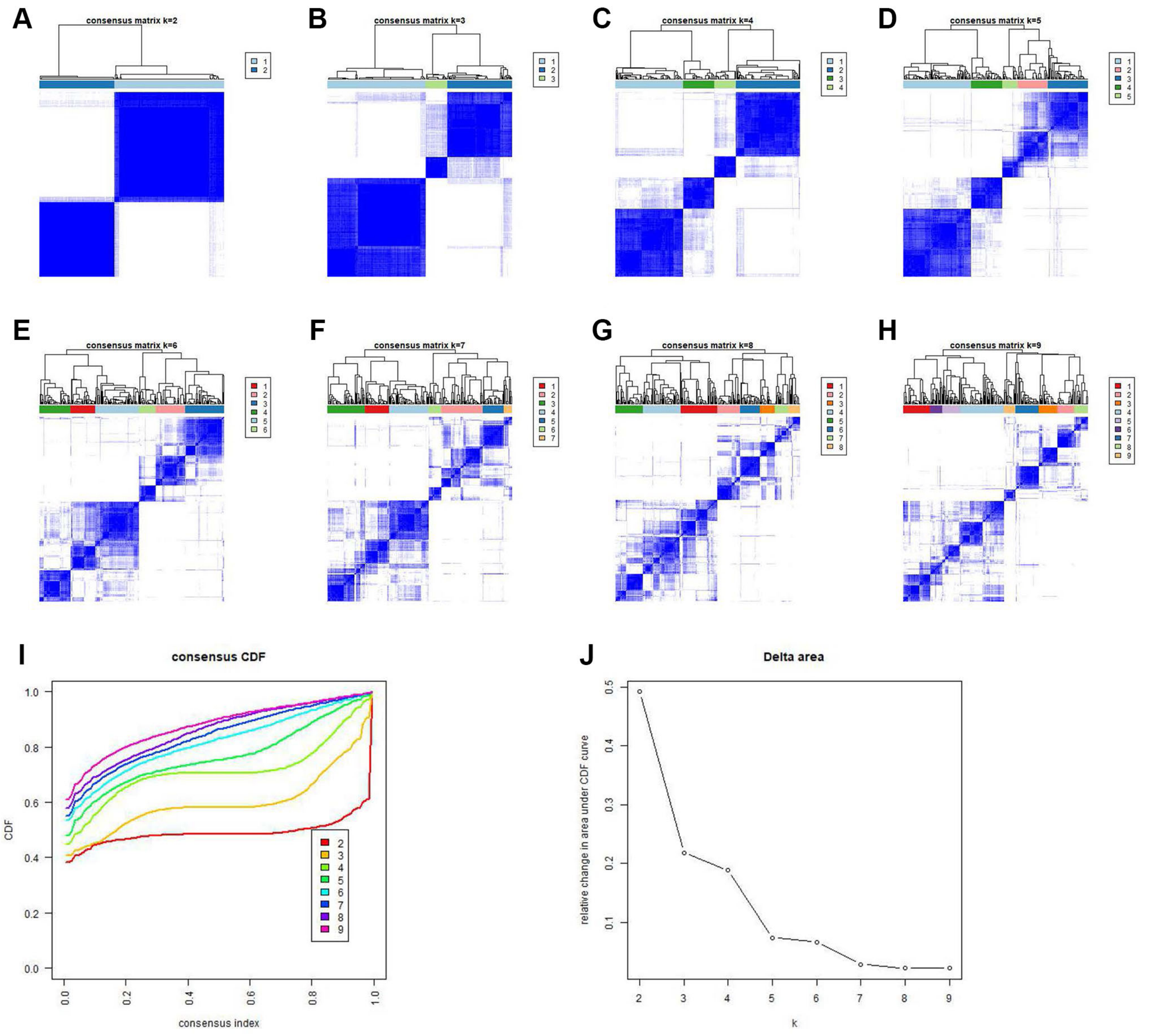

Supplementary Figure 3. Consensus clustering analysis of m^{6}A modification clusters. (**A**–**H**) Consensus matrices of the patients with sarcoma for k = 2–9. (**I**) Cumulative distribution function (CDF) of consensus clustering analysis. (**J**) Relative change in area under the CDF curve of consensus clustering analysis.

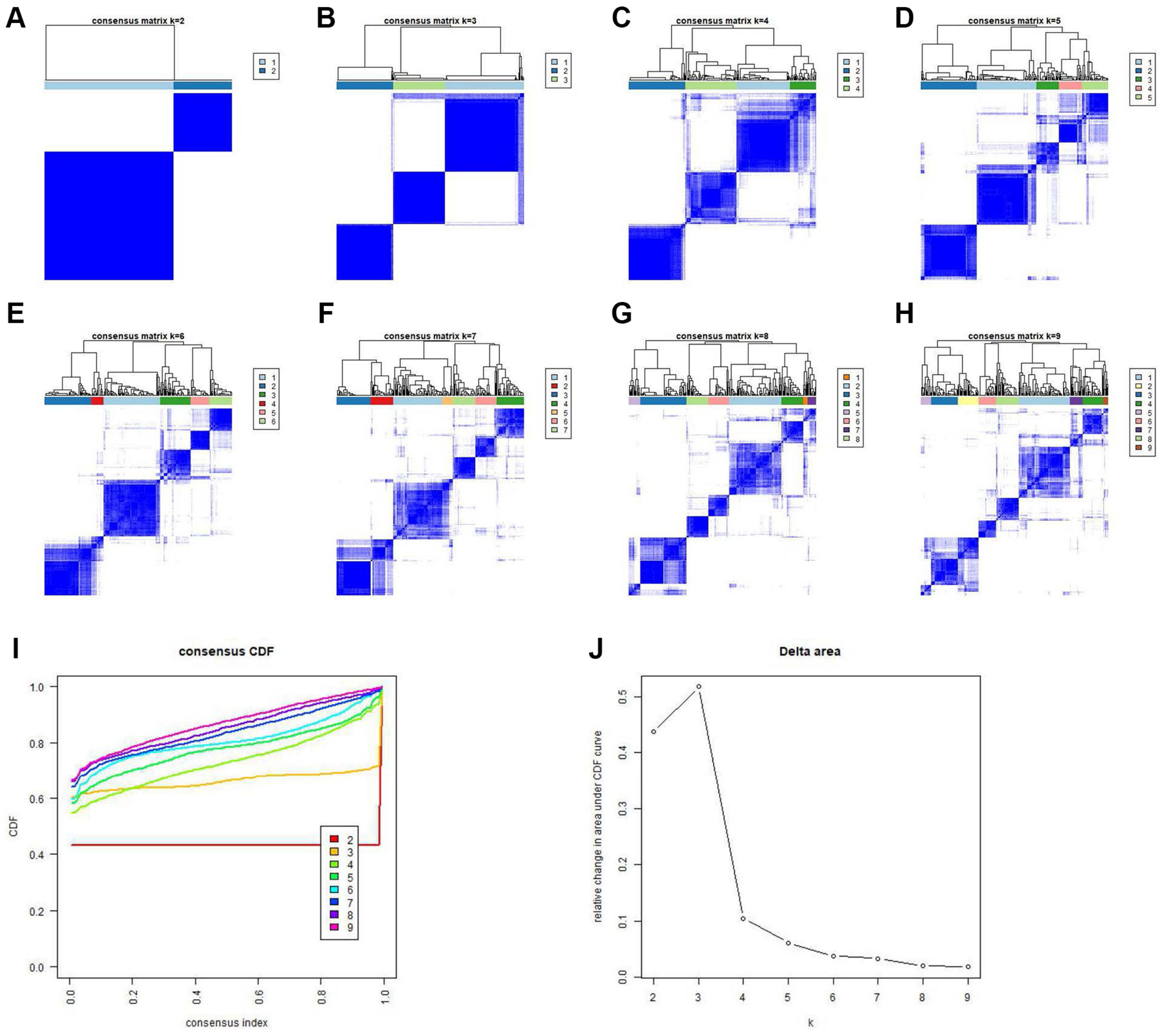

Supplementary Figure 4. Consensus clustering analysis of m^{6}A gene clusters. (**A**–**H**) Consensus matrices of the patients with sarcoma for k = 2–9. (**I**) CDF of consensus clustering analysis. (**J**) Relative change in area under the CDF curve of consensus clustering analysis.

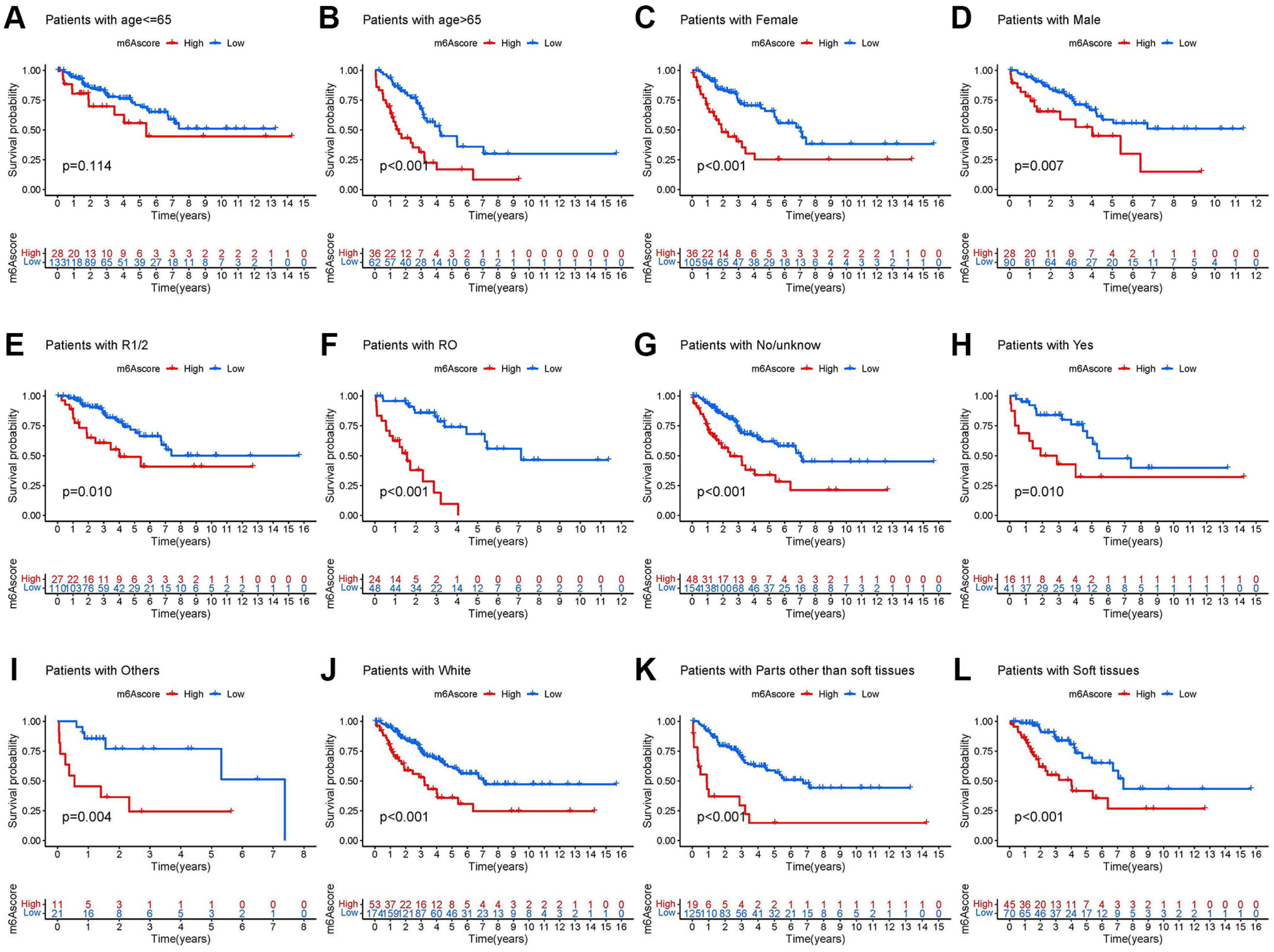

Supplementary Figure 5. Kaplan-Meier (K-M) analysis of different clinicopathological features in high- and low-m^{6}A score groups. (**A**) Age (age ≤ 65); (**B**) Age (age > 65); (**C**) Gender (Female); (**D**) Gender (Male); (**E**) Margin (R1/2); (**F**) Margin (RO); (**G**) Metastasis (No/unknown); (**H**) Metastasis (Yes); (**I**) Race (Others); (**J**) Race (White); (**K**) Site (Parts other than soft tissues); (**L**) Site (Soft tissues).
