# Supplementary material for: m6A regulator-mediated methylation modification patterns and tumor immune microenvironment in sarcoma
Source: Aging (Albany NY). 2022 Jan 3;14(1):330–53. doi: 10.18632/aging.203807 (PMC8791212; doi:10.18632/aging.203807)
Supplement: Supplementary Tables 1-3 [file aging-14-203807-s002.pdf]

SUPPLEMENTARY TABLES

Supplementary Table 1. The 23 N^6-methyladenosine (m^{6}A) regulators and their types.

Gene	Type
CBLL1	writers
VIRMA	writers
METTL14	writers
METTL3	writers
RBM15	writers
RBM15B	writers
WTAP	writers
ZC3H13	writers
ELAVL1	readers
FMR1	readers
HNRNPA2B1	readers
HNRNPC	readers
IGF2BP1	readers
IGF2BP2	readers
IGF2BP3	readers
LRPPRC	readers
YTHDC1	readers
YTHDC2	readers
YTHDF1	readers
YTHDF2	readers
YTHDF3	readers
FTO	erasers
ALKBH5	erasers

Supplementary Table 2. Univariate Cox regression analysis between 23 m^{6}A regulators and the prognosis of patients with sarcoma.

Genes	HR	HR.95L	HR.95H	*p* value
CBLL1	0.987946497	0.96792828	1.00837872	0.245608968
VIRMA	1.020245477	1.001055583	1.039803235	0.038558877
METTL14	0.984210143	0.960471466	1.008535538	0.201366338
METTL3	1.009449577	0.991025738	1.028215927	0.316945737
RBM15	1.012546871	0.989893507	1.035718648	0.280111444
RBM15B	1.019962902	0.99921603	1.041140545	0.059407543
WTAP	0.99611343	0.977705061	1.014868394	0.682411136
ZC3H13	0.997945483	0.983712034	1.012384878	0.779018426
ELAVL1	1.021702847	0.996193527	1.047865379	0.096045309
FMR1	1.006913884	0.991195559	1.02288147	0.390717979
HNRNPA2B1	1.029067753	1.009173243	1.049354457	0.004018014
HNRNPC	1.028329687	1.006938317	1.050175495	0.009197266
IGF2BP1	1.01752744	1.007412385	1.027744057	0.000652553

IGF2BP2	1.007904652	1.002903766	1.012930475	0.001918887
IGF2BP3	1.014359157	1.004393692	1.024423498	0.004650716
LRPPRC	1.010975473	0.994962862	1.027245785	0.18023504
YTHDC1	1.011754942	0.987901228	1.036184624	0.337048627
YTHDC2	0.998674934	0.97826206	1.019513752	0.899858998
YTHDF1	1.010100437	0.990100876	1.03050398	0.324650043
YTHDF2	1.041110126	1.019054692	1.063642907	0.000226265
YTHDF3	1.009027431	0.992122686	1.026220217	0.297164378
FTO	0.993242582	0.976943695	1.009813393	0.421874008
ALKBH5	0.9962828	0.986223642	1.006444558	0.471974814

Supplementary Table 3. Kaplan-Meier (K–M) analysis between 23 m^{6}A regulators and the prognosis of patients with sarcoma.

Genes	K–M value
METTL3	0.010691281
METTL14	0.007747145
METTL16	0.141020305
WTAP	0.034642704
VIRMA	0.0694909
ZC3H13	0.007800758
RBM15	0.113802444
RBM15B	0.077648902
YTHDC1	0.001146628
YTHDC2	0.025401174
YTHDF1	3.49E-06
YTHDF2	0.002707548
YTHDF3	0.010291272
HNRNPC	0.000119071
FMR1	0.000262172
LRPPRC	0.083835289
HNRNPA2B1	0.037667126
IGFBP1	0.187384629
IGFBP2	0.086013948
IGFBP3	2.86E-07
RBMX	0.031859669
FTO	0.037951415
ALKBH5	0.043621219
