# Supplementary material for: m6A regulator-mediated methylation modification patterns and tumor immune microenvironment in sarcoma
Source: Aging (Albany NY). 2022 Jan 3;14(1):330–53. doi: 10.18632/aging.203807 (PMC8791212; doi:10.18632/aging.203807)
Supplement: Supplementary Tables 5 and 6 [file aging-14-203807-s004.pdf]

SUPPLEMENTARY TABLES

Supplementary Table 5. DEG intersections.

Gene ID
CDK6
EZH1
POPDC2
FILIP1
IL6R
TP53INP2
TAGLN
HMGA1
LINC00888
SEMA7A
RUNX2
HSPB8
MYOM1
AOC3
ZHX2
LIMS2
PRKAG2
ARHGEF10L
SH3BGRL
PARM1
A2M
C11orf96

Supplementary Table 6. Univariate Cox regression analysis of 19 prognosis-related DEGs.

ID	HR	HR.95L	HR.95H	*p* value
CDK6	1.009792144	1.002383	1.017256	0.009498
EZH1	0.986785685	0.973965	0.999775	0.046194
POPDC2	0.992457193	0.987348	0.997593	0.004039
FILIP1	0.991284505	0.983833	0.998793	0.022984
IL6R	0.984817779	0.975454	0.994271	0.001698
TP53INP2	0.989839095	0.981187	0.998568	0.022609
TAGLN	0.995780277	0.992804	0.998765	0.005618
HMGA1	1.007732345	1.00311	1.012376	0.001024
SEMA7A	1.006572593	1.001704	1.011465	0.008094
RUNX2	1.006835934	1.001232	1.012471	0.016733
HSPB8	0.993486625	0.989581	0.997408	0.001148
AOC3	0.992256118	0.987973	0.996558	0.000428
ZHX2	0.98915193	0.979309	0.999094	0.032545
LIMS2	0.986175928	0.980104	0.992286	9.99E-06
PRKAG2	0.986008251	0.976491	0.995619	0.00441
SH3BGRL	0.991622516	0.983443	0.99987	0.046517
PARM1	0.994161576	0.989296	0.999051	0.019313
A2M	0.992624042	0.98729	0.997987	0.007087
C11orf96	0.993273644	0.988734	0.997834	0.003879
